# Retracted: Immunopathology and Immunogenetics of Allergic Bronchopulmonary Aspergillosis

**DOI:** 10.1155/2015/213928

**Published:** 2015-09-07

**Authors:** Journal of Allergy

The paper titled “Immunopathology and Immunogenetics of Allergic Bronchopulmonary Aspergillosis” [1], published in Journal of Allergy, has been retracted as it was found to contain a substantial amount of material from the following published article: “Allergic bronchopulmonary aspergillosis in asthma and cystic fibrosis” published in Clinical and Developmental Immunology, vol. 2011, Article ID 843763, 13 pages, 2011, DOI: 10.1155/2011/843763.
